# Geophysical and geochemical study of the contaminant impact of Oke-Tage solid waste dumpsite, Southwestern Nigeria

**DOI:** 10.1038/s41598-023-31948-3

**Published:** 2023-03-22

**Authors:** Michael Oluwatosin Adedinni, Augustine Babatunde Arogundade, Odunayo Timothy Ore, Charles Itunu Adenika, Adebiyi Samuel Adebayo, Grace Olubunmi Akinlade, Musa Olufemi Awoyemi, John Adekunle Oyedele Oyekunle

**Affiliations:** 1 Department of Physics and Engineering Physics, Obafemi Awolowo University, Ile-Ife, Nigeria; 2 Department of Chemistry, Obafemi Awolowo University, Ile-Ife, Nigeria; 3 Department of Basic Science- Physics, Babcock University, Ilishan Remo, Nigeria; 4 Department of Physics, University of Medical Sciences, Ondo State, Ondo, Nigeria

**Keywords:** Environmental sciences, Environmental chemistry, Environmental impact

## Abstract

The physicochemical properties of groundwater, geochemical characteristics and subsurface formation of the Oke-Tage waste dumpsite soil material were assessed to determine the impact of the leachate generated from the waste dumpsite on the quality of the groundwater within the study area. Water samples collected from hand-dug wells were analyzed to determine groundwater quality, while soil samples were examined for their geochemical characteristics. Ten Vertical Electrical Sounding (VES) surveys were carried out with an electrode spacing (AB/2) increasing from 1 to 200 m. Also, four 2D electrical resistivity profilings were done using the dipole–dipole configuration. The hydro-chemical analysis showed an elevated Cadmium (Cd) and Lead (Pb) concentration above the maximum permissible limits. The physicochemical results indicated that the Electrical Conductivity (EC) ranged from 1900 to 3670 µS/m, while Total Dissolved Solid (TDS) ranged from 585 to 620 mg/L. The health risk assessment showed no significant health risks associated with exposure to the metals due to HI values less than 1. Based on the VES result, four geoelectric layers comprising topsoil, weathered layer, fractured basement, and fresh basement were identified. The 2D resistivity structures revealed that the topsoil and weathered layers practically merged and are characterized by relatively low resistivity (< 30 Ωm) beneath the dump site. The study concluded that the groundwater and soil in the vicinity of the investigated Oke-Tage waste dumpsite had been negatively impacted to levels that called for caution especially using the water for regular potability purposes.

## Introduction

Waste is linked to virtually every aspect of human activity and is generated from our daily activities. It results from an event or process that does not have immediate economic value or demand and must be discarded^1^. Solid waste is undesirable materials produced in a given area, such as residential, industrial, or commercial activities. It could be classified by origin (domestic, agricultural, retail, building, or institutional), substance (organic material, glass, steel, plastic, and paper), or potential hazard (toxic, non-toxic, flammable, radioactive, or inflective)^2^.

The continuous increase in the urban population increases the quantity of waste generated in those areas^3^. Solid waste undergoes slow aerobic and anaerobic decompositions over the years. It produces substantial amounts of leachate with decomposing products such as landfill gas, heavy metals, and varieties of hazardous contaminations that may flow into underground aquifers from the landfill site. The management of solid wastes is a global environmental concern, especially in developing countries such as Nigeria^4^. In Nigeria and other developing countries, a common practice is to employ the use of open dumpsites for the disposal of municipal solid wastes^5,6^. The predominant challenge associated with the management of these sites includes weak policies made by the government^4^.

Leachate could be described as a liquid or water-soluble compound in the dumpsite generated through the decomposition of waste. This water-soluble matter is redistributed in the environment due to runoff resulting from rainfall or wet precipitation. Leachate may migrate from the dumpsite and seep into the soil to contaminate soil and groundwater in a way that poses a challenge to human health and the environment^7–9^. The factors that affect the generation of leachate include climate (rainfall), topography (run-on/runoff), and vegetation^10^.

The Oke-Tage waste dumpsite currently being investigated has been active for over two decades. The dumpsite hosts various types of waste, such as garbage, paper, plastic, glass, metal scrap, and expired drugs. Some of these wastes occur as homogeneous materials that are primarily non-biodegradable and have been compacted over the years. These allowed long-time interaction among the dumpsite materials, soil, and the subsurface geological system. Wastes deposited into dumpsites undergo oxidation, corrosion of metallic components, and decomposition of organic matter, resulting in the generation and release of leachate, which can impact the soil surface and groundwater resources, thereby affecting groundwater resources^11^. Waste materials that are over twenty years old are expected to biodegrade and produce effluent. The effluent generated by percolation may have found its way into the groundwater. However, there is the possibility that the leachate generated from the dumpsite may have impacted the immediate environment.

Research works such as^12–24^ have evaluated the impact of waste dumpsites on the environment using various techniques. However, the use of traditional sampling and drilling may not suffice in a detailed mapping of municipal wastes. This is because a complete assessment of municipal waste sites involves the consideration of several factors including waste composition, surface–subsurface geological conditions, and hydrological features among others^25^. Hence, the need arises to employ the use of geophysical and geochemical methods for a comprehensive evaluation of the environmental impacts of refuse dumpsites^26^. The geochemical and geophysical methods are essential in evaluating and characterizing contaminants generated by urban residues for environmental impact assessment. These contaminants are due to the occurrence of potentially toxic elements and other ions at refuse dumpsites and a probable transfer of these contaminants to nonpolluted areas, thereby posing adverse health effects to the local populace. Some of the potential human health effects accrued upon exposure to contaminated water include chest pains, tetanus, dengue fever, diarrhoea, skin infections, cholera, dysentery, typhoid, and eczema^27^.

Although significant research has been carried out on the contamination levels of refuse dumpsites across the globe, this study represents the first comprehensive attempt at characterizing the impacts of refuse dumpsites on the soil and groundwater of the study area (Oke-Tage) via the simultaneous use of geophysical and geochemical methods. The present study is designed to evaluate the potential impacts of open refuse dumpsites on the potable water resources as well as the agricultural soils of the study area for the purpose of environmental management and sustainability. The study addresses the need to achieve sustainable development goals comprising good health and well-being as well as clean water and sanitation.

### Description and geological setting of the study area

**Figure 1:**
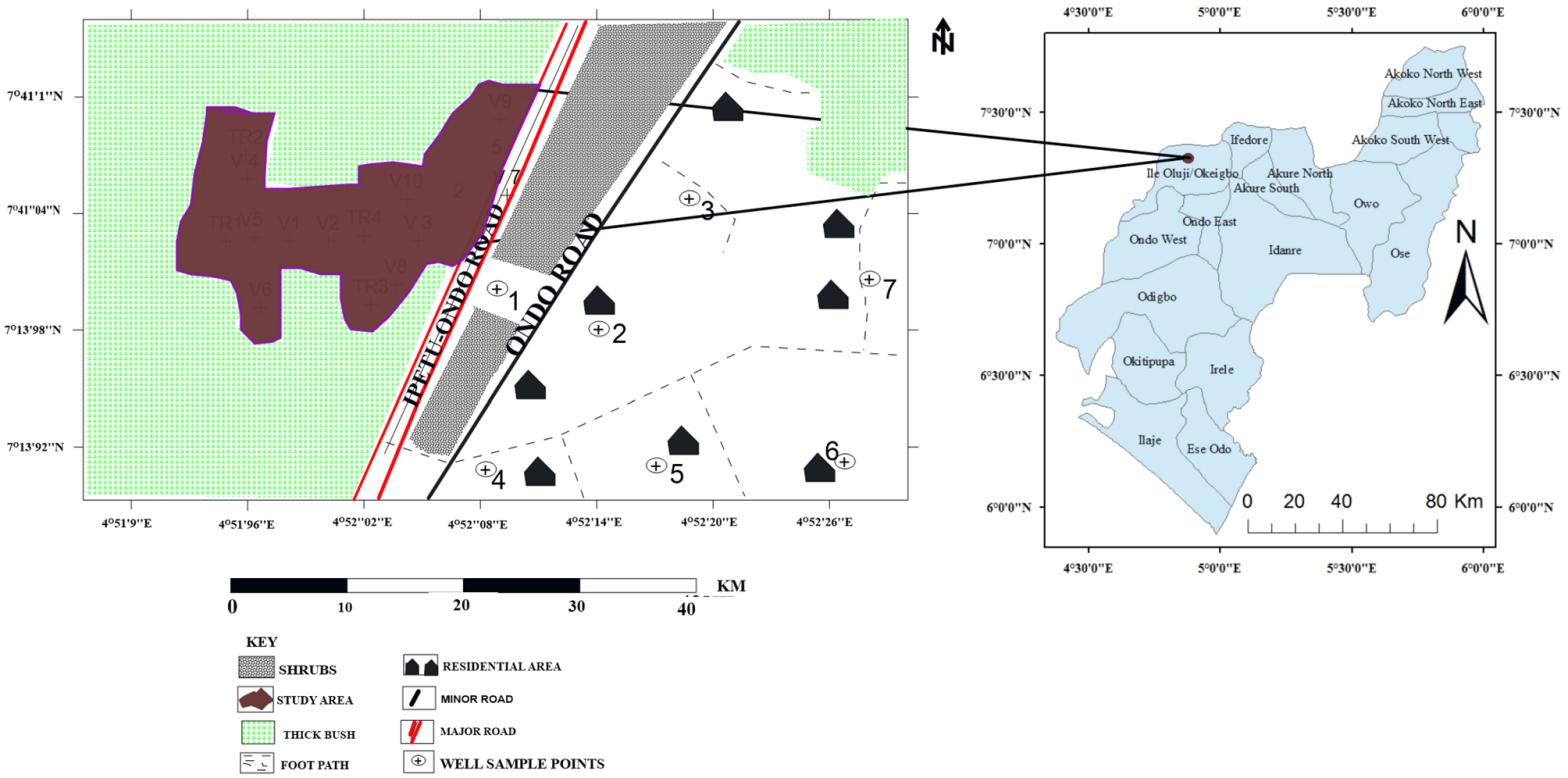
The Map of Ondo State showing the study location.

The Oke-Tage dumpsite is located on a 10 hectares landmark along Ondo-Ipetu-Ijesa road in Ile-Oluji Town, Ile-Oluji/Okegbo Local Government, Ondo State (Fig. 1).

The terrain within the area is relatively flat and accessible by footpaths and roads. The climatic condition of the study area is similar to the general climate of Southwestern Nigeria, a lowland tropical rainforest type with distinct wet and dry seasons. In the southern part of Ondo State, the mean monthly temperature is about 21 °C with a mean monthly range of 29 °C, while the mean relative humidity is over 75%. The mean monthly humidity is about 65%^28^. The solid waste from surrounding industries, schools and residential areas forms the solid composition of the dumpsite.

The study area is located within the Basement Complex terrain of Ondo State, Southwestern Nigeria. The local lithological units identified in the study area are granites, quartzite, and migmatite-gneiss. Quartzite is widely spread in the area, covering over half of the area, while granite-gneiss occurs as intrusive, low-lying outcrops in the Northeastern and Southeastern parts of the area. Quartzite ridges occur in several locations, mainly in the central part of the study area. The underlying basement rocks, which are concealed, may contain extensively faulted and folded areas, developing joint systems, and fracture systems that have resulted from multiple tectonic events^29,30^. In tropical and equatorial regions, weathering processes create surface layers with varying levels of porosity and permeability. Research has indicated that if the unconsolidated overburden is sufficiently thick, it could potentially serve as a dependable aquifer.

## Method of study

### Soil chemical analyses

**Figure 2:**
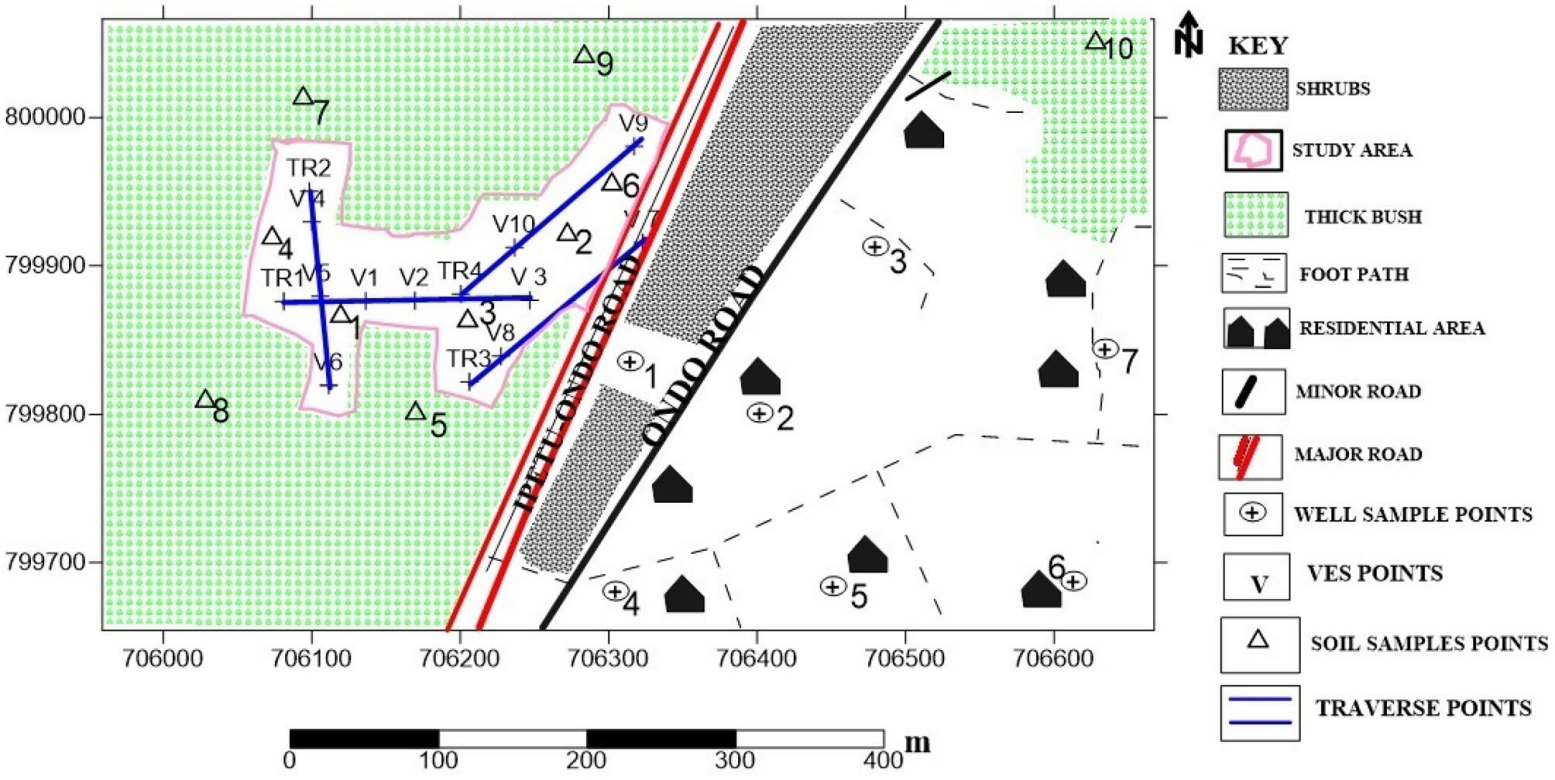
Data Acquisition Map of Oke-Tage Dumpsite.

Chemical analysis was conducted on the soil samples obtained within and outside the dumpsite to obtain a preliminary estimate of the level of contamination. Ten soil samples were collected randomly at 0.5 m depth from the study area using a hand auger. The soil samples were put in airtight polymer material and labelled. Soil samples S_1_ to S_5_ were taken within the dump site. Samples S_6_ to S_9_ were taken at a distance radius of about 50 to 200 m to the dump site. Sample S_10_ was taken at a distance of 700 m, far away from the dumpsite, to serve as a control sample (Fig. 2). The soil samples were analyzed for anions and cations.

### Hydro‑chemical analyses

The general information on water samples obtained from the artesian wells in the study area is presented in Table 1. The table gives the details of the sample description, longitudes, latitudes, elevations, and distances from the centre of the dump site. Seven artesian well water samples were collected within the study area and analyzed for various physical and chemical properties. Six water samples were collected at a distance that ranged from 367 to 513 m from hand-dug wells, while the seventh water sample was taken at a distance of 667 m away from the dump site to serve as a control sample.

**Table 1:** General information on water sample location.

Sample Code	Longitude (East)	Latitude (North)	Elevation (m)	Distance from centre of dumpsite (m)
WELL 1	4° 52′ 34.4″	7° 13′ 01.2″	240	367
WELL 2	4° 52′ 33.1″	7° 13′ 03.0″	245	375
WELL 3	4° 52′ 31.1″	7° 13′ 05.4″	249	387
WELL 4	4° 52′ 29.9″	7° 13′ 58.3″	252	433
WELL 5	4° 52′ 31.3″	7° 13′ 56.7″	255	467
WELL 6	4° 52′ 3.12″	7° 13′ 56,8″	250	513
WELL 7	4° 52′ 48.9″	7° 13′ 09.7″	251	667

### Sample digestion and analysis

One gram of each of the soil samples and 20 mL of each of the water samples was weighed and digested with a mixture of HCl and HNO_3_ (aqua regia) in a ratio of 1:3 using a temperature-controlled hotplate at 70 °C under a fume cupboard. The heating continued until digestion was completed. The digest was allowed to cool for some minutes and then transferred into a 50 mL standard volumetric flask. The digested samples were taken for elemental analysis using Atomic Absorption Spectrophotometer (Model PG 90).

### Geophysical survey

The electrical resistivity method was adopted for the survey because of its response to water-bearing materials, indicating the resistivity and conductive nature of underground layers. The survey was carried out close to and away from the dump site. At each station, four electrodes were arranged collinearly and Vertical Electrical Sounding (VES) using the 1D Schlumberger technique was carried out at electrode spacing (AB/2) increasing from 1 to 200 m. The acquired data was plotted as VES curves with the aid of bi-logarithmic papers by plotting the apparent resistivity (ρ_a_) against electrode separation and interpreted using the conventional partial curve matching technique with the aid of master curves and auxiliary point charts to obtain initial geoelectric parameters (resistivity and thickness) values of several geoelectric layers at respective VES stations. The obtained geoelectric parameters (layers resistivity and thicknesses) were used for iterative computer modelling using the WinResist software version 1.0^31,32^. Four 2D electrical resistivity profiling was carried out in the study area using the dipole–dipole electrical configuration. The results obtained from the 2D survey were converted into 2D resistivity structures with DIPROFWIN Version 4.0 software^33^. The interpreted results from the geophysical investigations were used to determine the subsurface lithology sequences and determine the lateral and the depth extents of contamination.

### Health risk assessment

The health risks associated with exposure to the potentially toxic elements in the studied soils via ingestion, inhalation, and dermal exposure were calculated using the formula below:

$${\text{CDI}}\;ingestion = \frac{{{\text{Cm }} \times {\text{ R}}ing{ } \times {\text{ EF }} \times {\text{ ED}}}}{{\text{ABW X AVT}}} \times 10^{ - 6}$$

$${\text{CDI}}\;inhalation = \frac{{{\text{Cm }} \times {\text{ R}}inh{ } \times {\text{ EF }} \times {\text{ ED}}}}{{{\text{ABW }} \times {\text{ AVT }} \times {\text{ PEF}}}}$$

$${\text{CDI}}\;dermal = \frac{{{\text{Cm }} \times {\text{ ESSA }} \times {\text{ SAF }} \times {\text{ DAF }} \times {\text{ EF }} \times {\text{ ED}}}}{{{\text{ABW }} \times {\text{ AVT}}}} \times 10^{ - 6}$$

$$HQ = \frac{CDI}{{RfD}}$$

$$HI = \sum HQ_{ing} + HQ_{inh} + HQ_{dermal}$$

All parameters are as defined by Adebiyi et al.^34^.

### Quality control/quality assurance protocols

For quality control, blank determinations were carried out. The reagents used for the sample digestion were taken through the digestion protocol without the samples. Recovery analysis was carried out by spiking 1 g of the soil samples with a 20 mg/kg standard mixture of the metal salts. The standard metal solutions were used to fortify the samples, digested, and then taken for AAS analysis. The percentage recovery was determined using the formula:

$$\% R = \frac{A - B}{C} \times 100$$

where the parameters are as defined by Adebiyi et al.^35^.

The reliability of the adopted analytical procedures in the present study was verified in terms of linearity of calibration (R^2^) and percentage recoveries (%R) of the metals which are listed in Table S1 (supplementary material). The percentage recoveries of the metals ranged from 84% in Mn to 99% in Zn indicating quantitative agreement in the recovery values. According to European Union guidelines^36^, the accuracy and precision of an adopted procedure are ascertained if the recovery values lie between 70 and 110%. The recovery values of the present study were within the certified range. The standard calibration curves of the instrument used (atomic absorption spectrophotometer) to determine the metal concentrations indicated high linearity levels with the R^2^ values ranging from 0.9665 for Cd to 0.9981 for Fe. These values indicated that the instrument can be relied on to give precise and accurate determinations of metal concentrations in the studied samples.

## Results and discussion

### Physicochemical analysis results

The results of the physicochemical analysis of the water samples are presented in Table 2. These were compared with World Health Organization and Nigerian Standard for Drinking Water Quality standard permissible limits.

**Table 2:** Result of physico-chemical analysis of the well water samples.

Parameter	Well 1	Well 2	Well 3	Well 4	Well 5	Well 6	Well 7 control	Range (Mean ± SD)	NSDWQ guideline	WHO guideline
Colour	Light brown	Colourless	Colourless	Colourless	Colourless	Colourless	Colourless	NA	Colourless	Colourless
Odour	Odourless	Mild	Odourless	Odourless	Odourless	Odourless	Odourless	NA	Odourless	Odourless
Temperature (^o^C)	28.3	27.5	28.4	27.6	27.6	27.3	27.1	27.1–28.4 (27.68 ± 0.48)	NA	25
pH	6.9	6.9	6.8	6.9	6.9	6.9	6.9	6.8–6.9 (6.88 ± 0.03)	7.0	7.5
TSS (mg/L)	280	270	265	280	221	221	98	98–280 (233.57 ± 64.96)	NA	NA
TDS (mg/L)	610	620	605	589	592	585	482	482–620 (583.28 + 46.37)	500	600
EC (µS/cm)	3670	2010	2101	1890	3650	1900	1250	1250–3670 (2353 ± 933.99)	NA	1500

SD = Standard Deviation, NA = Not Available, TSS = Total Suspended Solid, TDS = Total Dissolved Solid, EC = Electrical Conductivity, NSDWQ = Nigerian Standard for Drinking Water Quality and WHO = World Health Organization.

The temperature and pH values of the water samples ranged from 27.1 to 28.4 °C and 6.8 to 6.9, respectively. The temperature and pH values were within the NSDWQ and WHO standard permissible limits, as shown in Table 2. The Electrical Conductivity (EC) ranged from 1900 to 3670 µS/cm, and Total Dissolved Solids (TDS) ranged from 585 to 620 mg/L.

The values of EC and TDS from wells 1 to 6 exceeded the required standard permissible limit of NSDWQ and WHO, and they were more than that of the control sample (well 7). The control sample had a value that was within the standard permissible limits (Table 2). The elevated EC and TDS values in wells 1–6 showed that the wells nearby of the investigated dumpsite had been considerably contaminated. The impurities could directly influence the drinking quality of water and affect the properties such as odour, colour, and taste^37^. The high EC values indicated that the high salt content of the well water samples might probably have emanated from the municipal leachate from the dumpsite. All the EC values from wells 1 to 6 were above the recommended permissible limits (Table 2). Therefore, water from wells 1 to 6 might not be suitable for regular drinking and domestic purposes. Other effects of high EC values include disturbance of salt and water balance, adverse effects on heart patients, and high blood pressure^38^. The elevated TDS indicated that the dumpsite was the main contributor of dissolved solids in the wells since all the values of the TDS from wells 1–6 exceeded the recommended permissible limits for drinking and domestic use (Table 2). A high concentration of TDS decreases palatability and may cause gastrointestinal irritation in humans and might also have a laxative effect^38^. According to the concentration of EC and TDS, water from wells 1 to 6 is not fit for human consumption. The results of the concentrations of cations and anions analyzed in the well water samples are presented in Table 3. These were compared with the WHO and NSDWQ standard permissible limits. The concentration of cadmium (Cd), lead (Pb), and iron (Fe) ranged from 0.060 to 0.117 mg/L, 0.012 to 0.110 mg/L, and 0.005 to 0.123 mg/L respectively. These values were far above the maximum permissible limits of 0.003 mg/L for Cd, 0.01 mg/L for Pb, and 0.02 mg/L for Fe.

**Table 3:** Hydro-chemical analysis of the well water samples.

Parameter (mg/L)	Well 1	Well 2	Well 3	Well 4	Well 5	Well 6	Well 7 control	Range (Mean ± SD)	NSDWQ	WHO
Cu	0.108	0.47	0.023	0.024	1.037	0.008	0.004	0.004–1.037 (0.239 ± 0.389)	1.0	2.0
Cd	0.117	0.112	0.022	0.051	0.078	0.023	0.006	0.006–0.117 (0.058 ± 0.044)	0.003	0.003
Pb	0.095	0.110	0.032	0.033	0.038	0.048	0.012	0.012–0.110 (0.052 ± 0.036)	0.01	0.01
Zn	0.063	0.057	1.026	0.032	0.048	0.011	0.008	0.008–1.026 (0.177 ± 0.374)	3.0	3.0
Fe	0.102	0.123	0.071	0.048	0.098	0.018	0.005	0.005–0.123 (0.066 ± 0.044)	0.3	0.3
Mn	0.104	0.046	0.021	0.029	0.036	0.007	0.004	0.002–0.104 (0.035 ± 0.033)	0.2	0.4
Mg	0.117	0.112	0.012	0.050	0.078	0.013	0.010	0.010–0.117 (0.056 ± 0.047)	20	0.05
K	0.089	0.104	0.037	0.038	0.042	0.052	0.028	0.028–0.089 (0.056 ± 0.029)	NA	12
Na	0.063	0.057	0.026	0.031	0.049	0.009	0.010	0.009–0.063 (0.035 ± 0.021)	200	200
Ca	0.104	0.125	0.069	0.059	0.097	0.001	0.017	0.001–0.104 (0.067 ± 0.045)	1.0	75
Ni	0.079	0.068	0.020	0.013	0.022	0.021	0.021	0.013–0.079 (0.035 ± 0.026)	0.02	0.07
Chloride	21.679	23.104	21.435	25.538	19.539	17.116	16.118	16.118–25.538 (20.647 ± 3.312)	250	NA
Phosphate	1.415	1.829	1.446	1.794	0.490	1.224	11.277	0.489–11.277 (2.782 ± 3.772)	NA	NA
Sulphate	20.195	19.568	22.989	18.202	22.810	20.760	18.550	18.203–22.989 (20.439 ± 1.898)	100	100
Nitrate	6.688	6.767	6.421	5.429	6.691	7.335	7.802	5.429–7.802 (6.733 ± 0.742)	50	50

SD = Standard Deviation; NA = Not Available; NSDWQ = Nigerian Standard for Drinking Water Quality. WHO = World Health Organization.

The source of the high concentration of elements (Cd, Pb, and Fe) in the wells could probably be due to the leachate generated from the dumpsite or used storage batteries dumped indiscriminately around the environment as observed in part of the Oke-Tage environs. The concentration of Cd, Pb, and Fe found in this study indicated that water from wells 1 to 6 is not safe for human consumption because elevated levels of Cd and Pb could cause adverse health effects such as renal disease and cancer problems to consumers^39^. Excessive iron levels in drinking water could lead to the early onset of skin wrinkles, haemochromatosis, clogging of pipes, and a metallic taste in food and drinks^40,41^.

The concentration of anions is generally below the standard permissible limit, and some of the cations also fell below the standard permissible limits. This observation implies that the dumpsite contributed minimally to the anions and some cations levels in the well water. The sources of these anions and cations might include industrial waste or emissions, agricultural runoff, car, and truck exhausts.

### Geochemical results

**Figure 3:**
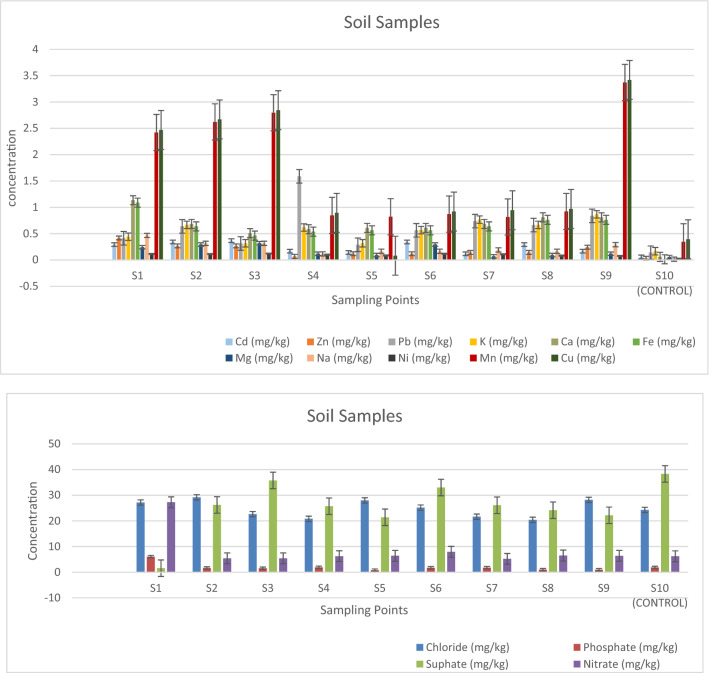
(**a**) Variation Plot of Heavy Metals Concentration across Sampling Points. (**b**) Variation Plot of Anions Concentration across Sampling Points.

The results of the geochemical analysis of the soil involving the concentrations of the metals and anions considered in this study are shown in Fig. 3a,b.

The results were compared with the soil control sample (S_10_). In consonance with the one-way analysis of variance (Table S2, supplementary material), it was discovered that the total metal concentrations obtained in the soil samples were comparatively higher than the total metal concentrations obtained in the control sample (*p* < 0.05) except for Cu, Zn, and Ni. These indicated a degree of contamination arising from anthropogenic activities within the Oke-Tage waste dump site.

The comparison of the concentrations of the total metal in the control sample (S_10_) with the standard permissible limits set by the Department of Petroleum Resources (DPR) indicated that the concentration of the metals was all below the standard permissible limits (Table 4). This indicated that the control sample (S_10_) was practically uncontaminated from anthropogenic source(s). The soils within the dumpsite are rich in potassium due to the impact of the dumpsite and possibly by the application of NPK fertilizer by farmers on the surrounding farmland. The concentrations of Pb obtained in all the soil samples were higher than the concentration of the control sample. Direct contact with Pb-contaminated soils may pose a human health risk ^42–44^ as it has no known essential function in the human body. It can also result in a wide range of biological effects depending on the level and duration of exposure.

**Table 4:** Comparison of the control sample values with the standard permissible limits.

Element	Control sample (mg/kg)	Target value (mg/kg) (DPR)
Cu	0.394	36
Zn	0.032	140
Cd	0.058	0.8
Mn	0.345	850
Pb	0.138	85
Fe	0.013	5000
Ni	0.023	35
K*	0.167	85
Ca*	0.059	260

*IUGS/IAGC Standard permissible limits.

In general, plants like tomatoes, peppers, melons, and okra do not accumulate large amounts of lead (Pb) from the soil. In contrast, some plants (e.g., beans, groundnut, soya beans, and cowpeas) may have a high affinity for Pb in contaminated soils^37^. In another case, soil particles containing lead (Pb) may cling to the outer leaves or the surfaces of the root of vegetables and might pose a health risk if eaten^39^. Cadmium (Cd) is highly toxic. A high concentration of Cd in the soil for the cultivation of vegetables and other food crops could lead to adverse health effects such as renal disease and cancer when consumed^39^. Also, direct exposure to elevated levels of Cd causes proximal tubular disease^45^. The result also shows that sample three (S_3_) had the highest concentration of Cd in the soil, and crops growing within the area might accumulate elevated Cd levels.

Therefore, soils in and around the dumpsite are not suitable for agricultural purposes as they might pose health challenges to humans and the general biota. Copper is an essential element, but a high dose can cause intestinal irritation and anaemia with direct contact^46^. Ni also can be dangerous when the maximum tolerable amounts are exceeded^46^. Other analyzed metals are Ca, Zn, Fe, Mg, Na, and Mn. The mean concentration values of the elements were more significant than the values of the control sample (S_10_) (Fig. 3a). All the elements are potentially toxic to plants and animals in large amounts^44^. The mean concentration values of the metals in the sampling points considered are within the intervention metal limits set by DPR.

The anion concentrations of the soil samples are presented in Fig. 3b. The mean concentration values of Nitrate (NO_3_^−^), Sulphate (SO_4_^2−^), Phosphate (PO_4_^3−^), and Chloride (Cl^−^) are 8.491 mg/kg, 24.017 mg/kg, 2.142 mg/kg, and 28.812, mg/kg respectively. A high concentration was recorded for NO_3_^-^ in S_1,_ while others were significantly lower than the S_1_ and the control sample (S_10_). This implied that the increase in NO_3_^−^ in S_1_ originated mainly from the dumpsite due to leachate because areas with high organic matter such as dump sites can release NO_3_^-^ through the activity of nitrifying bacteria in the soil^39^. Crops planted around this area will grow slower than in other areas, and algal growth will be stimulated in the well water, leading to a eutrophic condition. Also, surface and groundwater within the study area might subsequently receive nitrate contents, rendering them unsuitable for human consumption.

All the samples indicated high content of Cl^−^ except for S_4_ and S_8_, with low concentrations compared to the control sample (S_10_). The high concentrations of Cl^−^ might become a limiting factor for plant growth as plants grown on soils with high Cl^−^ content might vittle or become stunted^47^. The observation shows that the dumpsite is not the primary source of Cl^-^ but probably contributed some Cl^−^ to the study area.

Analytical results showed that low concentrations of SO_4_^2−^ were observed compared with the control sample, except for sample S_1,_ which is significantly lower than others. The result revealed that other sources apart from the dumpsite could influence the concentration of SO_4_^2-^ in that area, such as industrial emissions or waste.

The phosphate concentration (PO_4_^3−^) indicated a high level was recorded in sample S_1_ while the concentration in other samples was lower than the values obtained in control sample S_10_. This observation suggested that the decrease in PO_4_^3−^ levels from samples S_2_ to S_9_ might be due to the surface runoff. This observation showed that the dumpsite was not the primary source of the PO_4_^3−^ to the study area, but probably it contributed very little concentration to the PO_4_^3−^ level in the study area. The range of contaminant concentrations and the physical and chemical forms will depend on activities and disposal patterns of the contaminated waste on the dumpsite.

The non-carcinogenic health risk assessment emanating from exposure to the potentially toxic elements in the soils around the studied refuse dumpsite via ingestion, inhalation, and dermal contact is presented in Table 5.

**Table 5:** Non-carcinogenic health risk assessment of potentially toxic elements in soils around Oke-Tage refuse dumpsite.

Elements	Population	CDI_(ing)_	CDI_(inh)_	CDI_(derm)_	RfD_(ing)_	RfD_(inh)_	RfD_(derm)_	HQ_(ing)_	HQ_(inh)_	HQ_(derm)_	HI
Cd	Children	3.16E-05	8.87E-11	8.86E-08	1.00E-03	1.00E-03	1.40E-01	3.16E-02	8.88E-08	6.33E-07	3.16E-02
	Adults	1.45E-06	1.36E-11	5.79E-08				1.45E-03	1.37E-08	4.14E-07	1.45E-03
Cu	Children	2.16E-04	6.06E-10	6.04E-07	4.00E-02	4.02E-02	2.40E-02	5.40E-03	1.51E-08	2.52E-05	5.43E-03
	Adults	9.91E-06	9.33E-11	3.95E-07				2.48E-04	2.32E-09	1.65E-05	2.64E-04
Fe	Children	8.54E-05	2.39E-10	2.39E-07	7.00E-01	NA	1.40E-01	1.22E-04	0.00E + 00	1.71E-06	1.24E-04
	Adults	3.92E-06	3.69E-11	1.56E-07				5.61E-06	0.00E + 00	1.12E-06	6.73E-06
Mn	Children	2.19E-04	6.17E-10	6.15E-07	1.40E-01	5.00E-05	1.84E-03	1.57E-03	1.23E-05	3.35E-04	1.92E-03
	Adults	1.01E-05	9.50E-11	4.02E-07				7.21E-05	1.90E-06	2.19E-04	2.93E-04
Ni	Children	1.31E-05	3.68E-11	3.68E-08	2.00E-02	2.06E-02	1.00E-03	6.57E-04	1.79E-09	3.68E-05	6.94E-04
	Adults	6.03E-07	5.68E-12	2.40E-08				3.02E-05	2.76E-10	2.41E-05	5.43E-05
Pb	Children	8.58E-05	2.40E-10	2.40E-07	4.00E-03	3.52E-03	5.25E-04	2.15E-02	6.84E-08	4.58E-04	2.19E-02
	Adults	3.94E-06	3.70E-11	1.57E-07				9.85E-04	1.05E-08	3.00E-04	1.28E-03
Zn	Children	2.53E-05	7.11E-11	7.10E-08	3.00E-01	3.00E-01	7.50E-02	8.46E-05	2.37E-10	9.47E-07	8.55E-05
	Adults	1.16E-06	1.09E-11	4.64E-08				3.88E-06	3.66E-11	6.20E-07	4.50E-06

CDI = chronic daily intake, RfD = reference dose, HQ = hazard quotient, HI = hazard index, ing = ingestion, inh = inhalation, derm = dermal.

The health risk assessment results indicated that ingestion was the principal exposure pathway to the potentially toxic elements, followed by dermal contact and inhalation. This is due to the increased chronic daily intake (CDI) and hazard quotient (HQ) values observed for the ingestion pathway. Frequent hand-to-mouth activity exhibited by children particularly might be a possible ingestion route for these potentially toxic elements^48^. Furthermore, children were observed to be more vulnerable to potentially toxic elements due to relatively high HQ values. This is consistent with the reports of previous findings^9,34,49^. The results of the hazard index (HI) showed that the HI values followed the order: Cd > Pb > Cu > Mn > Ni > Fe > Zn. This indicated that the highest risk of metal exposure was attributed to Cd. This finding was corroborated by earlier reports where Cd was implicated in posing the most potent health risks among studied metals^48,50^. Some possible health disorders widely associated with exposure to Cd include cellular DNA damage, neurological dysfunction, brain retardation, disrupted growth and development, liver damage, intestinal irritation, declined fertility, miscarriage, and impaired cognitive function ^51–53^. Although the concentrations of the potentially toxic elements are high in the studied soils, the calculated HI values are less than 1, indicating that the studied soils do not pose immediate adverse effects on the local population's health^54^.

### Geophysical results

#### Vertical electrical sounding (VES) result

**Figure 4:**
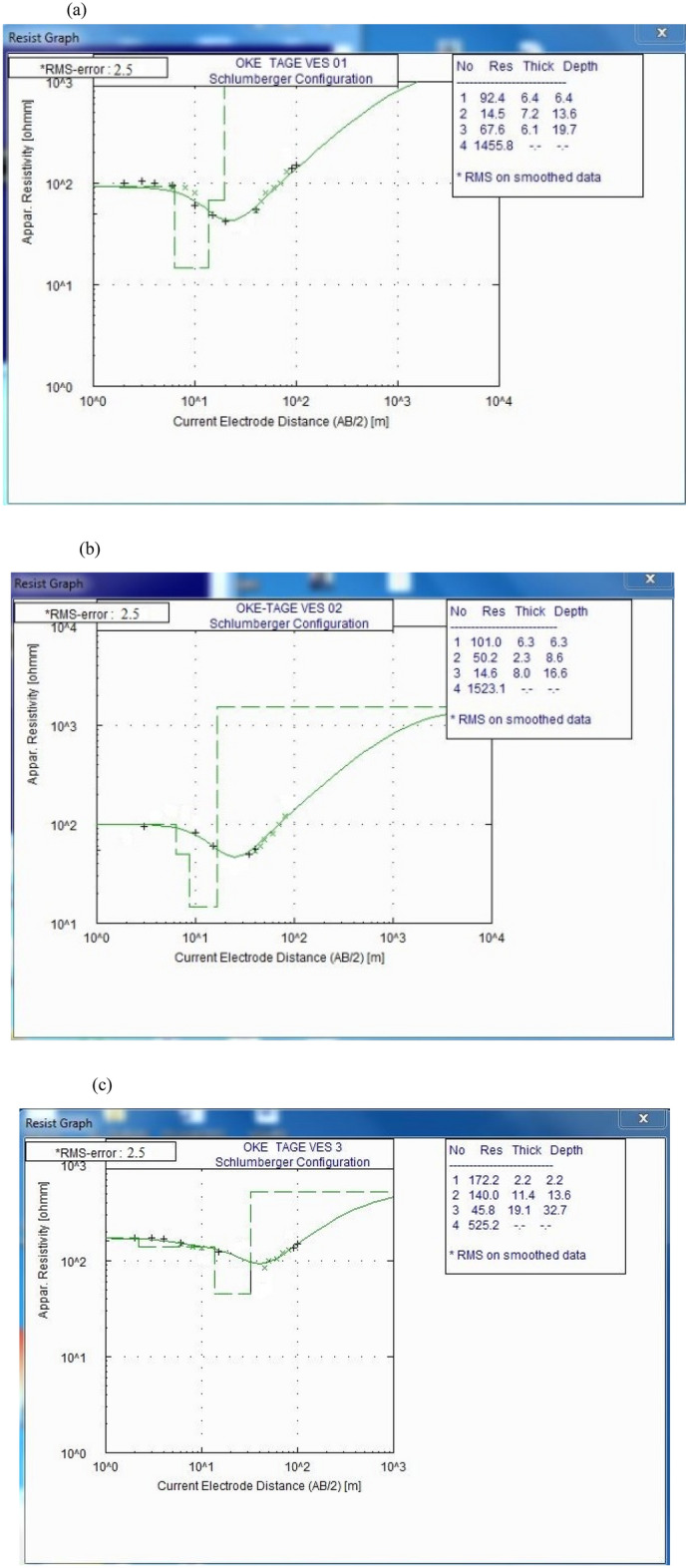
1D Model of (**a**) VES 1 (**b**) VES 2 (**c**) VES 3.

The field curve types obtained from the processed VES data are HK, HA, QH, and HKH shown in Fig. 4a–c.

The curve type HA is predominant with 40% occurrences, followed by HK with 30% occurrences. The QH and HKH type curve has 20% and 10% occurrences, respectively. The HA-type curve shows that the basement rock might have been subjected to minimal tectonic activity with a consequent low frequency of fracture in the basement and serves as an area of accumulation of leachate plume^55,56^. Table 6 shows the summary of the interpretation result and their lithologic unit classification. From the interpreted results of the VES curves, the 2D geoelectric sections show the resistivity variations and thickness of layers of each position within the depth penetrated in the study area. These VES sections were generated based on the iterated geoelectric parameters (layers resistivity and thicknesses).

**Table 6:** Summary of the interpretation results and their lithologic unit classification.

VES Number	Number of Layers	Resistivity Value (Ωm)	Thickness (m)	Depth (m)	Geological Implication	Curve type
1	1	92	6.4	6.4	Topsoil	HA
	2	15	7.2	13.6	Weathered layer	
	3	68	6.1	19.7	Weathered layer	
	4	1456	–	–	Fresh basement	
2	1	101	6.3	6.3	Topsoil	QH
	2	50	2.3	8.6	Weathered Layer	
	3	15	8.0	16.6	Weathered layer	
	4	1523	–	–	Fresh basement	
3	1	172	2.2	2.2	Topsoil	QH
	2	140	11.4	13.6	Clayey sand	
	3	46	19.1	32.7	Weathered layer	
	4	525	–	–	Fracture basement	
4	1	118	3.3	3.3	Topsoil	HK
	2	105	30.3	33.6	Weathered layer	
	3	469	49.0	83.0	Fracture basement	
	4	334	–	–	Fracture basement	
5	1	160	1.6	1.6	Topsoil	
	2	140	22.3	23.9	Weathered layer	HKH
	3	1340	45.8	69.7	Fresh basement	
	4	682	31.4	101.1	Fractured basement	
	5	4191	–	–	Fresh basement	
6	1	119	7.1	7.1	Topsoil	HA
	2	49	16.3	23.4	Weathered layer	
	3	192	8.2	31.6	Fracture basement	
	4	734	–	–	Fresh basement	
7	1	226	7.0	7.0	Topsoil	HK
	2	95	19.0	26.0	Weathered layer	
	3	567	33.0	59.0	Fracture basement	
	4	393	–	–	Fracture basement	
8	1	81	5.0	5.0	Topsoil	HA
	2	28	7.0	12.0	Weathered layer	
	3	504	23.0	35.0	Fracture basement	
	4	776	–	–	Fresh basement	
9	1	150	5.3	5.3	Topsoil	HK
	2	81	13.0	18.3	Weathered layer	
	3	578	24.0	42.3	Fracture basement	
	4	572	–	–	Fracture basement	
10	1	151	8.0	8.0	Topsoil	HA
	2	53	11.0	19.0	Weathered layer	
	3	1214	30.0	49.0	Fresh Basement	
	4	1338	–	–	Fresh basement	

**Figure 5:**
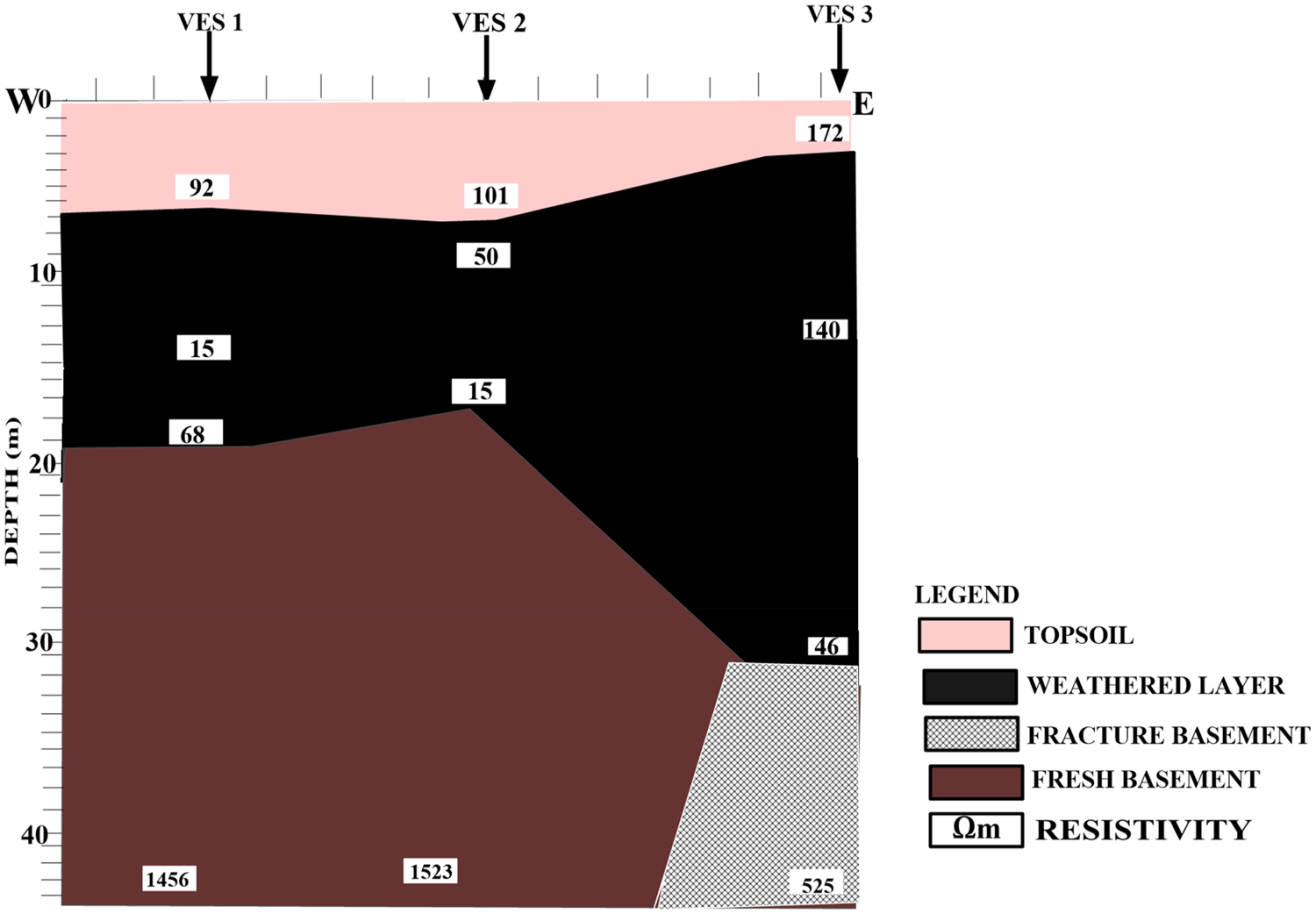
2D Geoelectric Section along W–E Cross Section.

Figure 5 shows a geoelectric section that relates VES stations 1, 2, and 3 along the W-E direction.

The profile revealed four subsurface layers: topsoil, weathered layer, fractured basement, and fresh basement. The first layer, topsoil, is characterized by resistivity ranging between 92 and 172 Ωm and has layer thickness ranging from 2.2 to 6.4 m. This indicates that the topsoil layer is a moderately permeable substrate that can act as a conduit for fluid flow. The second layer, is the weathered layer, of clayey sand (with layer resistivity ranging from 15 to 140 Ωm while the thickness ranges from 2.3 to 11.4 m). The relatively low resistivity values of 15 to 50 Ωm obtained from VES 1 and 2 beneath the dumpsite could be attributed to the effect of leachate from the dumpsite. The low resistivity variation indicates the degree of decomposition of the waste materials and saturated zones of leachate within the subsurface^12,16^. This may be a result of the vertically downward migration of leachate into the weathered layer. The third layer, also a weathered layer, is characterized by low resistivity values ranging from 15 to 68 Ωm. The mean resistivity of the third layer is 43 Ωm, and the layer thickness ranges from 6.1 to 19.1 m, with a mean thickness of 11.7 m. The low resistivity values and thickness of the lithologic units imply that layers 2 and 3 might be composed of low impermeable substratum and saturated with leachate^47^. The fourth layer shows fracture/fresh basement with resistivity values ranging from 1456 to 1523 Ωm, and the layer thickness varies from 20 to 32.0 m. The resistivity value of 525 Ωm in this layer beneath VES 3 is typical of a fractured basement that may serve as a zone for leachate accumulation and conduit to contaminate the groundwater^12^.

**Figure 6:**
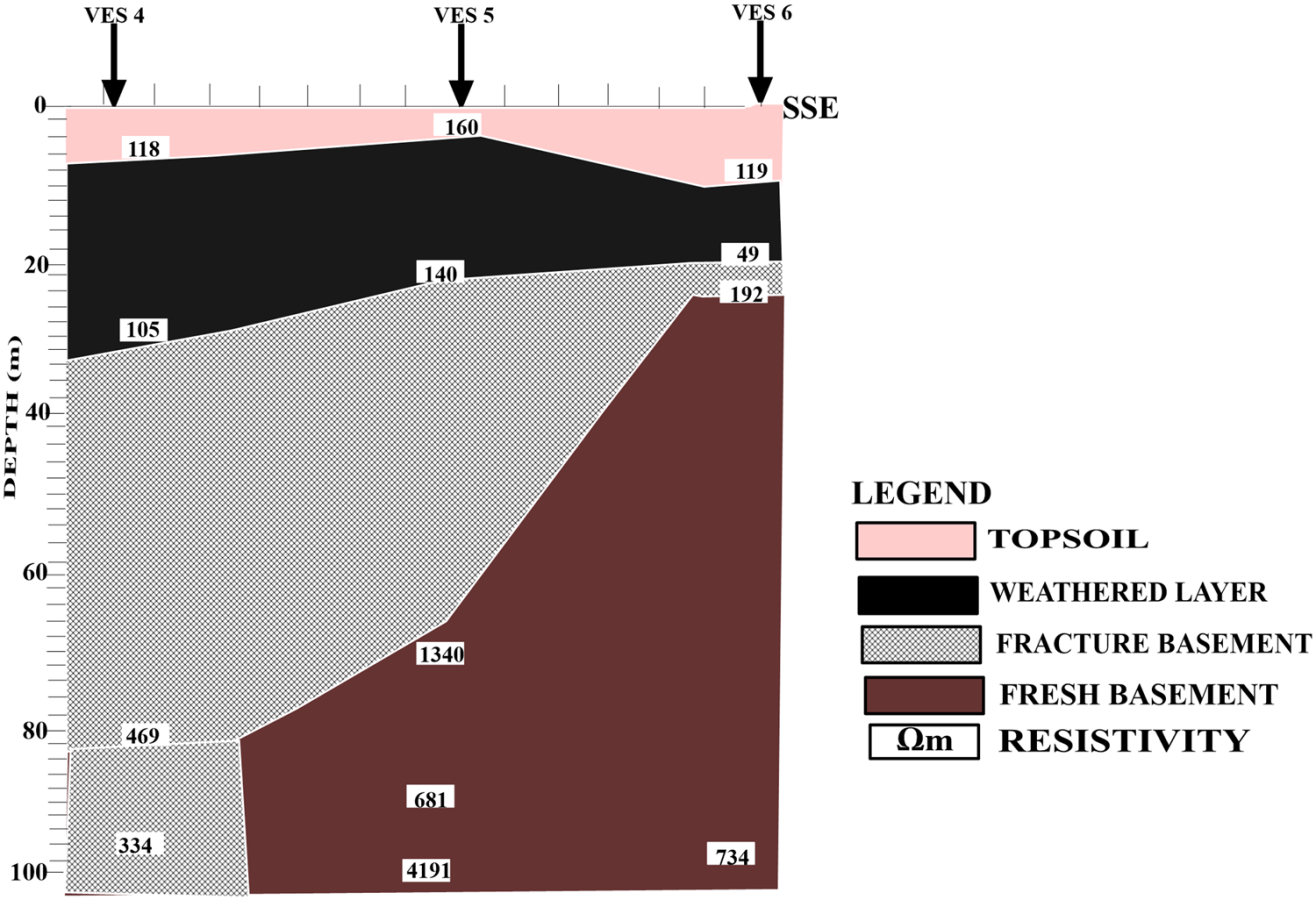
2D Geoelectric Section along NNW–SSE Cross Section.

Figure 6 shows a geoelectric section that relates VES stations 4, 5, and 6 along the N-S direction.

The profile has revealed the presence of four distinct subsurface layers. The first layer, which is the topsoil, has a resistivity range between 118 and 160 Ωm and a thickness ranging between 1.6 and 7.1 m. The topsoil layer is composed of sandy clay, indicating that it is porous, but the thickness of the layer implies that it will not allow significant vertical migration of contaminants. The second layer is the weathered layer, also composed of sandy clay, with resistivity values ranging from 49 to 140 Ωm and a thickness between 16.3 and 30.7 m. This layer is characterized by moderately low resistivity values, indicating it is porous and permeable, meaning it can easily allow leachate migration vertically downward from the strata above at or around the dump site. However, the thickness of the weathered layer implies that it will result in low-permeable leachate contaminating the groundwater. The third layer is the fractured basement, which has resistivity values ranging from 192 to 682 Ωm and a thickness ranging from 8.2 to 49 m. This layer is a potential pathway for groundwater contamination if the migration of contaminants from the overlying layers can migrate through to this layer. The fourth and deepest layer is the fresh basement, which has an undulating topography and resistivity values ranging from 734 to 4191 Ωm. The high resistivity values in this layer imply that it is composed of solid rock.

**Figure 7:**
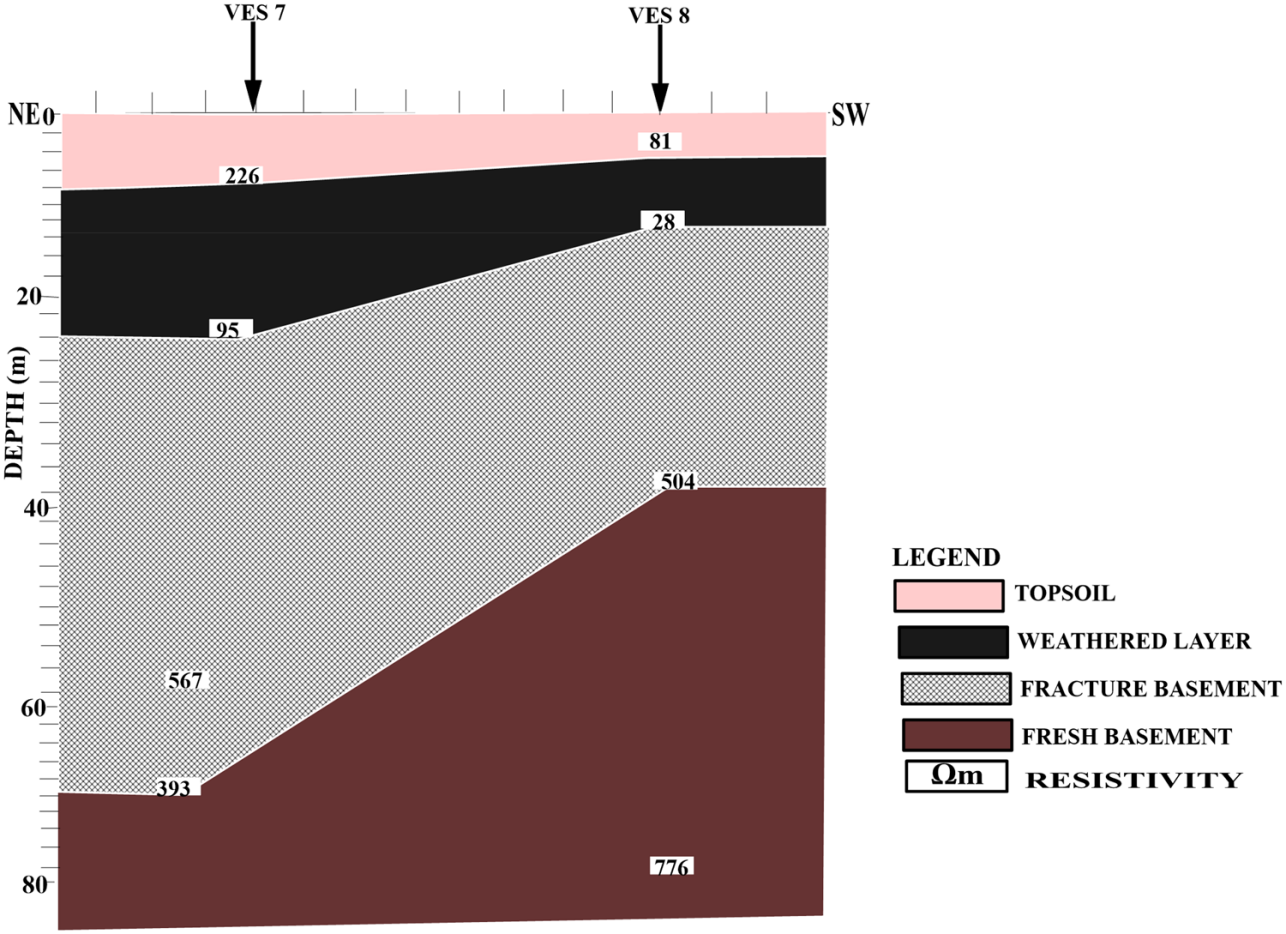
2D Geoelectric Section along NE–SW Cross Section.

Figure 7 shows a geoelectric section that relates VES stations 7 and 8 along NE–SW direction.

The profile revealed four subsurface layers: topsoil, weathered layer, fractured basement, and fresh basement. The topsoil layer around VES 8 has a resistivity range of 81–226 Ωm and a layer thickness range of 5.0–7.0 m. This layer is composed of sandy clay that has been contaminated with leachate, which could be attributed to the waste materials from the dump site. The presence of leachate indicates that the waste materials are decomposing, and there is a high potential for groundwater contamination in the area. The weathered layer has resistivity values ranging from 28 to 95 Ωm and thickness ranging from 7.0 to 19.0 m. The relatively low resistivity values of 28 to 95 Ωm obtained from VES 7 and 8 beneath the dumpsite could be attributed to migrating leachate from the decomposing wastes in the dumpsite. The fractured basement is characterized by resistivity values ranging from 393 to 567 Ωm with layer thickness ranging from 23.0 to 33.0 m. This layer may be indicative of the accumulation zone of leachate^16^ which poses a risk to groundwater quality. The fresh basement is characterized by a resistivity value of 776 Ωm and has an undulating basement relief. The resistivity value 393 Ωm observed beneath VES 7 may be suggestive of the leachate accumulation zone.

**Figure 8:**
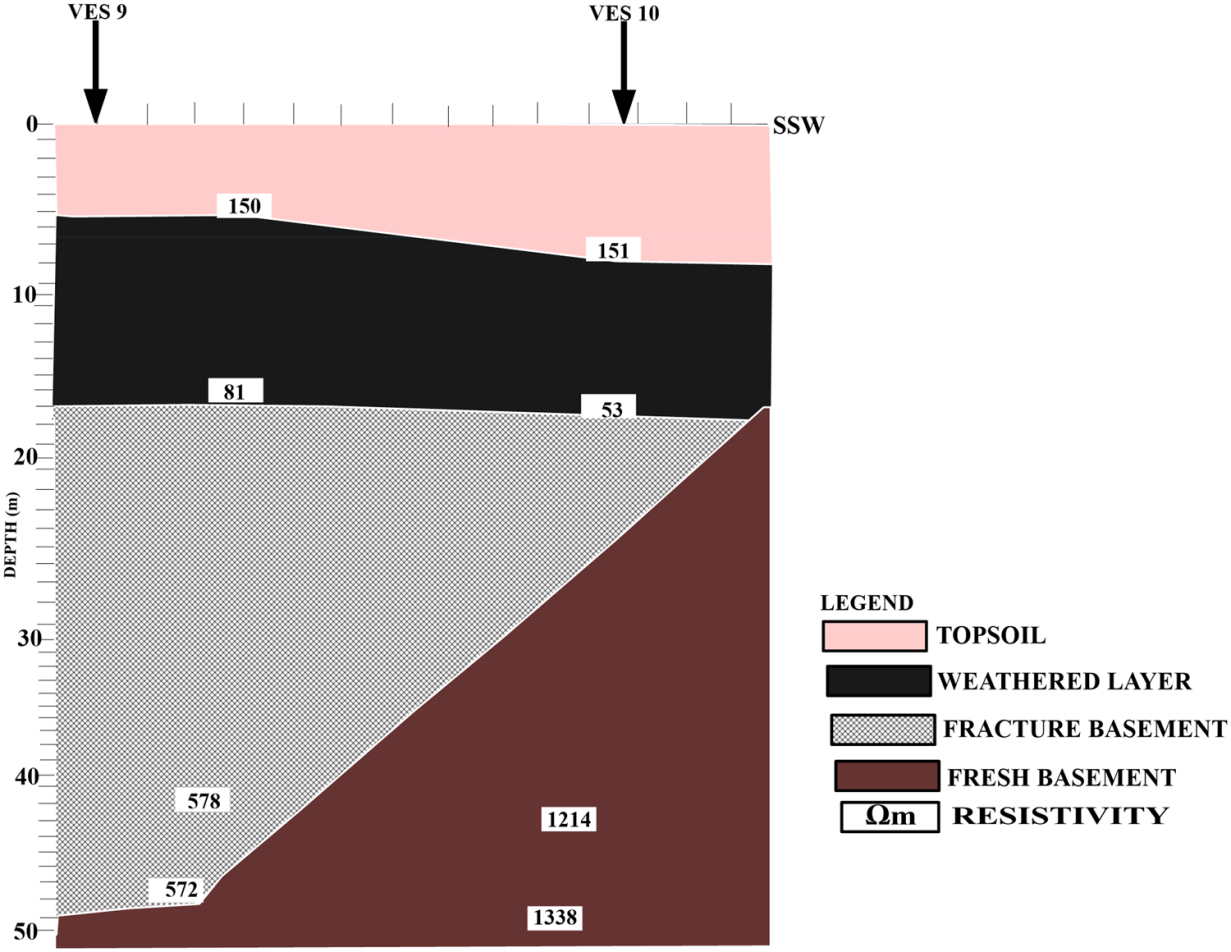
2D Geoelectric Section along NNE–SSW Cross Section.

Figure 8 shows a geoelectric section that relates VES stations 9 and 10 along NW–SE.

The profile shows four subsurface layers: topsoil, weathered layer, fractured basement, and fresh basement. The topsoil is characterized by resistivity ranging from 150 to 151 Ωm and layer thickness ranging from 5.3 to 8.0 m. The mean resistivity of the topsoil is 151 Ωm which implies that the topsoil is composed of sand contaminated by a leachate plume. The weathered layer is characterized by resistivity ranging from 53 to 81 Ωm with a mean resistivity of 67 Ωm and layer thickness ranging from 11.0 to 13.0 m with a mean thickness of 12.0 m. The mean resistivity indicates that the zone is highly contaminated by leachate. The zone constitutes clay soil that has low porosity. A fractured and fresh basement characterizes the third layer, with layer thickness ranging from 24.7 to 29.6 m. The resistivity values of the third layer ranged from 578 to 1214 Ωm with a mean of 896 Ωm. The mean resistivity simply implies an uncontaminated zone because of the low porosity from the weathered layer^16^. VES 10 extended to the fresh basement with a resistivity of 1214 Ωm. The fresh basement is characterized by resistivity ranging from 572 to 1338 Ωm, and it is unevenly distributed (undulating basement relief). This layer showed a resistivity value of 572 Ωm beneath VES 9, and it is categorized as a fractured basement. The fracture within the basement might serve as a conduit and zone for the accumulation of leachate seepage^47^.

The weathered and fractured basement can play an important role in contaminant transport. The permeability of the weathered layer depends on various factors such as the type of rock, the degree of weathering, and the presence of fractures or cracks within the layer. The presence of sandy clay in the weathered layer will enhance a good water retention capacity due to the clay particles, but its porosity is generally lower than that of sandy soils, making it less permeable to water and contaminant transport through the subsurface. A fractured basement, on the other hand, can act as a preferential pathway for contaminant migration due to the presence of interconnected cracks and fractures.

The thickness of the lithologic units is also important in contaminant transport. A thicker layer may provide more storage capacity for contaminants, which can result in a longer residence time in the subsurface and potentially increased mobility. Additionally, thicker layers can result in more complex flow patterns and interactions with other subsurface features such as aquifers, which can further affect contaminant transport. The thickness of the layers can also affect the rate of groundwater recharge, which can influence the movement of contaminants.

### Dipole–dipole data interpretation

**Figure 9:**
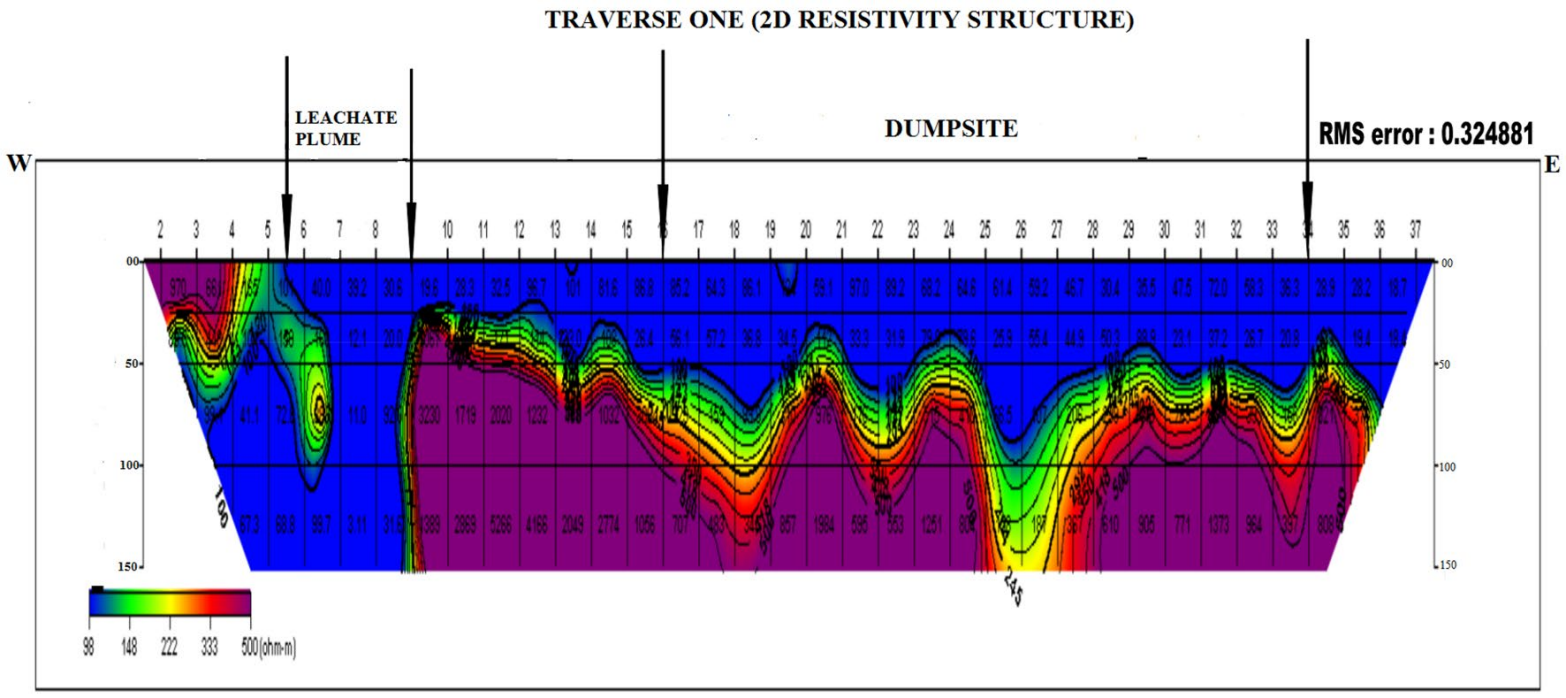
2D Resistivity Structure of Traverse 1.

**Figure 10:**
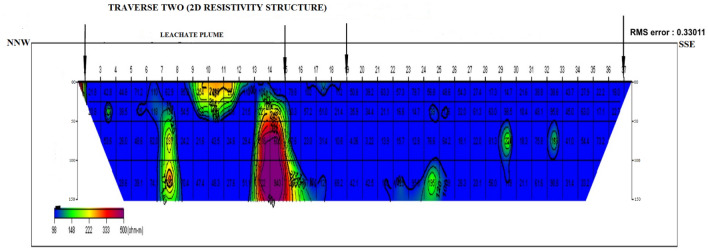
2D Resistivity Structure of Traverse 2.

**Figure 11:**
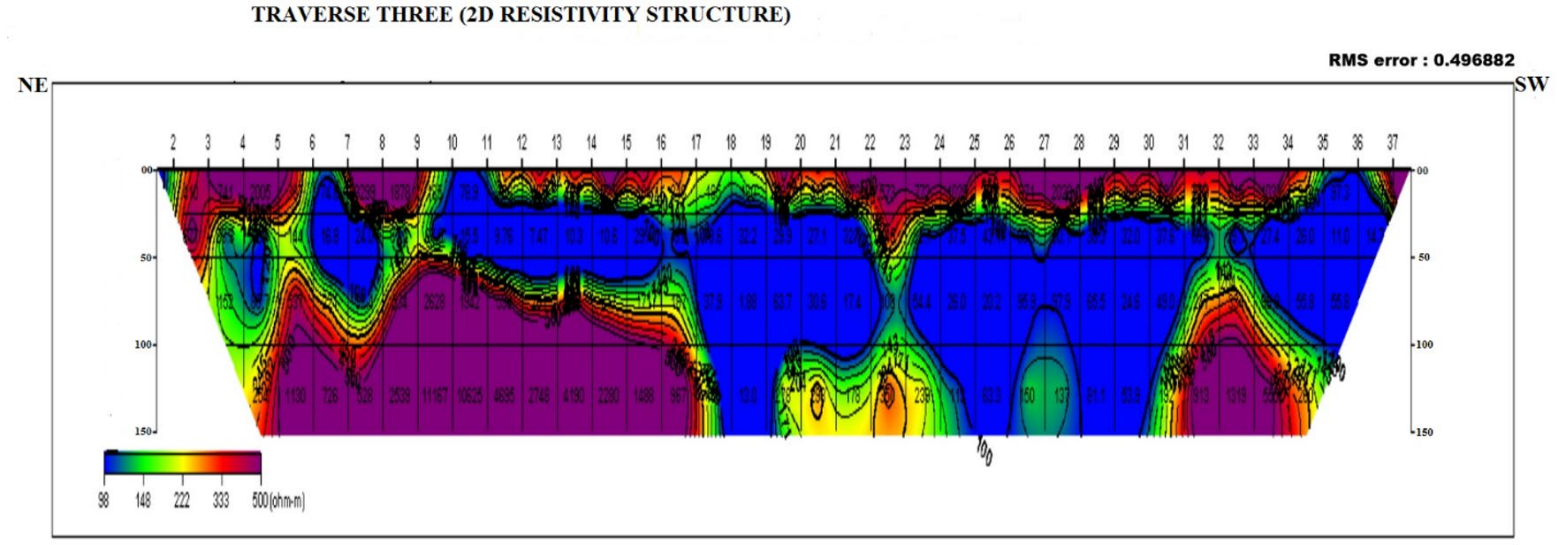
2D Resistivity Structure of Traverse 3.

**Figure 12:**
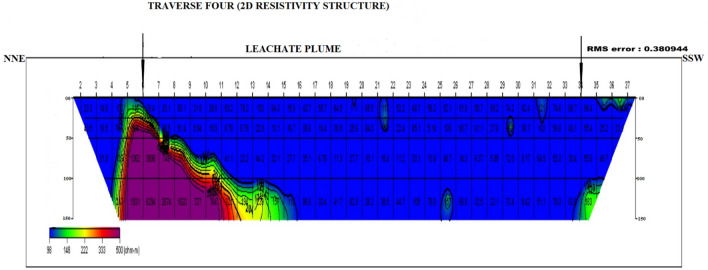
2D Resistivity Structure of Traverse 4.

Four dipole–dipole data profiles were carried out within and around the Oke-Tage waste dump site. The resistivity values < 100 Ω$\mathrm{m}$ in Figs. 9, 10, 11, 12 are depicted with a blue colour band.

It signifies a relatively low resistivity zone that has been contaminated. Resistivity values > 200 $\mathrm{\Omega m}$ with yellow, red, and purple colour bands indicate an uncontaminated zone^47^.

The 2D resistivity structure of transverse 1 shown in Fig. 9 extends to 200 m in the W-E direction towards the dump site. Between stations 5 and 37 along the profile, a very low resistivity is visible at a depth of 0 to 50 m. This low resistivity is associated with leachate contamination originating from the waste dumpsite. A decreased resistivity < 30  Ω$\mathrm{m}$ is observed between stations 6 and 9. This zone showed penetration of leachate plume that migrated within the dumpsite and the extent of leachate contamination was estimated to a depth > 150 m beneath the dump site.

Figure 10 shows the 2D resistivity structure of transverse two extending through to 200 m in the NNW-SSE direction to the dumpsite and a perpendicular direction to transverse 1. Within stations 2 and 16, resistivity values < 100 Ωm were observed at the topsoil. This low resistivity showed that the topsoil in this area is contaminated. A part along the profile showed that the topsoil has virtually merged with the weathered layer because of overlapping low resistivity values and a relatively small thickness. The soil within the area of the dumpsite is observed to have a resistivity < 50 Ωm. This may imply that the leachate plume originated between stations 3 and 8 and flowed to a depth > 150 m within the dumpsite area.

Figure 11 shows the 2D resistivity structure of transverse three, extending to 200 m along the NE-SW direction of the dumpsite. The weathered layer along the transverse between stations 17 and 36 at depths 50 m to 100 m showed consistently low resistivity < 70 Ω$\mathrm{m},$ which may indicate contamination by leachate plume due to its lateral migration from the dump site. As shown in Fig. 11, the leachate plume might have originated from stations 6 to 7 and stations 10 to 16 and flowed downward along with the weathered layer at a depth of 100 to 150 m towards the southeast direction of the dumpsite.

Figure 12 shows the 2D resistivity structure of transverse four along the NNE–SSW direction of the dumpsite. The profile had a total length of 200 m and fell directly on the margin of the dumpsite. The 2D resistivity structure indicated that the topsoil has a resistivity < 80 Ωm between stations 7 and 28, indicating the leachate contamination on the topsoil. The topsoil has virtually merged with the weathered layer because of the overlapping low resistivity values and relatively small thickness.

Areas where the topsoil and weathered layer practically merged and were characterized with low resistivity beneath the dumpsite were ascribed to leachate penetration around the dumpsite with a depth extent computed to be > 30 m. This may be due to the overburden relative permeability, probable linear features, and the descending slope of the bedrock topography near the dump site. Regions like the NNE—SSW and NNW—SSE part of the dumpsite were saturated with leachate and later migrated to relatively porous and permeable neighbouring substrata. This migration is considered slow because there was no trace of contamination 700 m away from the dump site.

## Conclusion

Combined geochemical and geophysical investigations were carried out within the Oke-Tage waste dumpsite, Ile-Oluji, Southwestern Nigeria. The relatively low resistivity of the weathered layer was suspected to be leachate saturation and hence, groundwater contamination around the dump site. The findings of the study have demonstrated that electrical resistivity imaging as well as geochemical analysis of soil and water samples can be useful tools in monitoring the extent of contamination of leachate plumes generated from refuse dumpsites. Although the health risk assessment showed no immediate significant health risks associated with exposure to the metals due to HI values less than 1, it is important to continuously monitor the levels of metals in the site to prevent biomagnification and bioaccumulation. Finally, the research revealed that the studied soil and water are contaminated with migrating leachate plumes within the study area. Therefore, there is a need to improve waste management practices to mitigate groundwater and soil contamination. It is suggested that continuous dumping of municipal solid wastes on the sites should be discouraged so that environmental sustainability is not threatened. Furthermore, policymakers and appropriate stakeholders should develop strategies that will ensure that the sites are restored to their pristine states in order to ensure a pollution-free environment.

## Supplementary Information

Supplementary Information.

## Data Availability

The datasets used and/or analyzed during the current study are available from the corresponding author upon reasonable request.

## References

[1] Debrah JK, Vidal DG, Dinis MAP (2021). Raising awareness on solid waste management through formal education for sustainability: A developing countries evidence review. Recycling.

[2] Saleh HM, Koller M (2019). Introductory chapter: Municipal solid waste. Munic. Solid Waste Manag..

[3] Akmal T, Jamil F (2021). Assessing health damages from improper disposal of solid waste in metropolitan Islamabad-Rawalpindi, Pakistan. Sustainability.

[4] Butt NA (2022). Geophysical and geochemical characterization of solidwaste dumpsite: A case study of Chowa Gujar, Peshawar (Part of Indus Basin). Sustainability.

[5] Iqbal MR, Hashimoto K, Tachibana S, Kawamoto K (2019). Geotechnical properties of sludge blended with crushed concrete and incineration ash. GEOMATE J..

[6] Ore OT (2019). Total and chemical speciation analyses of potential toxic metals in refuse dumpsite soils. World J. Appl. Chem..

[7] Hughes, K. L., Christy, A. D. & Heimlich, J. E. Landfill types and liner systems. *Ohio State University Extension Fact Sheet CDFS-138–05* (The Ohio State University, Columbus, OH, USA, 2008).

[8] Fetter CW, Boving T, Kreamer D (2017). Contaminant Hydrogeology.

[9] Ojo AO, Olurin OT, Ganiyu SA, Badmus BS, Idowu OA (2020). Hydro-geochemical assessment of an open dumpsite in a basement complex of Abeokuta, Ogun State, Southwestern Nigeria. Arab. J. Geosci..

[10] Alslaibi T (2009). Evaluating the Impact of Landfill Leachate on Groundwater Aquifer in Gaza Strip Using Modeling Approach.

[11] Olagunju E, Badmus O, Ogunlana F, Babalola M (2018). Environmental impact assessment of waste dumpsite using integrated geochemical and physico-chemical approach: A case study of Ilokun waste dumpsite, Ado-Ekiti, Southern Nigeria. Civ. Eng. Res. J..

[12] Bayode S, Omosuyi G, Mogaji K, Adebayo S (2011). Geoelectric delineation of structurally-controlled leachate plume around Otutubiosun dumpsite, Akure, Southwestern Nigeria. J. Emerg. Trends Eng. Appl. Sci..

[13] Nava-Martínez EC, Flores-García E, Espinoza-Gomez H, Wakida FT (2012). Heavy metals pollution in the soil of an irregular urban settlement built on a former dumpsite in the city of Tijuana, Mexico. Environ. Earth Sci..

[14] Ayolabi EA, Folorunso AF, Kayode OT (2013). Integrated geophysical and geochemical methods for environmental assessment of municipal dumpsite system. Int. J. Geosci..

[15] Ebistu TA, Minale AS (2013). Solid waste dumping site suitability analysis using geographic information system (GIS) and remote sensing for Bahir Dar Town, North Western Ethiopia. Afr. J. Environ. Sci. Technol..

[16] Adebayo A, Ariyibi E, Awoyemi M, Onyedim G (2015). Delineation of contamination plumes at Olubonku dumpsite using geophysical and geochemical approach at Ede Town, Southwestern Nigeria. Geosciences.

[17] Cınar H, Altundaş S, Ersoy E, Bak K, Bayrak N (2016). Application of two geophysical methods to characterize a former waste disposal site of the Trabzon-Moloz district in Turkey. Environ. Earth Sci..

[18] Joe-Ukairo A, Oni AG (2018). Geophysical and hydro-chemical investigations of Oke Asunle dumpsite in Ile-Ife, Southwestern Nigeria for subsoil and surface water pollution. J. Health Pollut..

[19] Mepaiyeda S, Baiyegunhi C, Madi K, Gwavava O (2019). A geophysical and hydro physico-chemical study of the contaminant impact of a solid waste landfill (swl) in King Williams’ Town, Eastern Cape, South Africa. Open Geosci..

[20] Alabi A (2020). Assessment of groundwater potential and quality using geophysical and physicochemical methods in the basement terrain of Southwestern, Nigeria. Environ. Earth Sci..

[21] Fatoba JO (2021). Geophysical and geochemical assessments of the environmental impact of Abule-Egba landfill, southwestern Nigeria. Model. Earth Syst. Environ..

[22] Okoro EE, Okolie AG, Sanni SE, Omeje M (2020). Toxicology of heavy metals to subsurface lithofacies and drillers during drilling of hydrocarbon wells. Sci. Rep..

[23] Kahal AY, Abdelrahman K, Alfaifi HJ, Qaysi S, Aldossari AN (2021). Geophysical assessment of open dumpsite nearby Khamis Mushait industrial zone, southwestern Saudi Arabia. J. King Saud Univ. Sci..

[24] Badmus G, Ogungbemi O, Enuiyin O, Adeyeye J, Ogunyemi A (2022). Delineation of leachate plume migration and appraisal of heavy metals in groundwater around Emirin dumpsite, Ado-Ekiti, Nigeria. Sci. Afr..

[25] El Maguiri A, Kissi B, Idrissi L, Souabi S (2016). Landfill site selection using GIS, remote sensing and multicriteria decision analysis: Case of the city of Mohammedia, Morocco. Bull. Eng. Geol. Env..

[26] Abou El-Magd I (2022). Qualitative and quantitative characterization of municipal waste in uncontrolled dumpsites and landfills using integrated remote sensing, geological and geophysical data: A case study. Sustainability.

[27] Babs-Shomoye F, Kabir R (2016). Health effects of solid waste disposal at a dumpsite on the surrounding human settlements. J. Public Health Dev. Ctries..

[28] Akinyemi S (2014). Mineralogy, physicochemical characteristics and industrial potential of some residual clay deposits within Ekiti state, southwestern Nigeria. J. Environ. Earth Sci..

[29] Mogaji K, Omosuyi G, Olayanju G (2011). Groundwater system evaluation and protective capacity of overburden material at Ile-olujI, Southwestern Nigeria. J. Geol. Min. Res..

[30] Olumuyiwa FO, Bamidele AM (2019). Groundwater resource assessment by hydraulic properties determination for sustainable planning and development in central Part of Ondo State, Nigeria. J. Water Sci. Environ. Technol..

[31] Vander Velpen B, Sporry R (1993). RESIST. A computer program to process resistivity sounding data on pc compatibles. Comput. Geosci..

[32] Obiora DN, Ibuot JC (2020). Geophysical assessment of aquifer vulnerability and management: A case study of University of Nigeria, Nsukka, Enugu State. Appl. Water Sci..

[33] Joel E (2019). Integration of aeromagnetic and electrical resistivity imaging for groundwater potential assessments of coastal plain sands area of Ado-Odo/Ota in southwest Nigeria. Groundw. Sustain. Dev..

[34] Adebiyi FM, Ore OT, Adegunwa AO, Akhigbe GE (2023). Source apportionment, health and ecological risk assessments of essential and toxic elements in kerosene-contaminated soils. Environ. Forensics.

[35] Adebiyi F, Ore O, Akhigbe G, Adegunwa A (2020). Metal fractionation in the soils around a refined petroleum products depot. Environ. Forensics.

[36] Commission E-E (2002). Directive (2002/32/EC) of the European Parliament and of the Council of 7 May 2002 on undesirable substances in animal feed. Off. J. Eur. Union C.

[37] Olafisoye ER, Sunmonu L, Adagunodo T, Oladejo OP (2013). Groundwater contaminats’ investigation at Aarada waste disposal site using geophysical and hydro-physicochemical approach. J. Environ. Sci. Toxicol. Food Technol. (IOSR-JESTFT).

[38] Akoto O, Gyamfi O, Darko G, Barnes VR (2017). Changes in water quality in the Owabi water treatment plant in Ghana. Appl. Water Sci..

[39] Gorenc S, Kostaschuk R, Chen Z (2004). Spatial variations in heavy metals on tidal flats in the Yangtze Estuary, China. Environ. Geol..

[40] Behera B, Das M, Rana G (2012). Studies on ground water pollution due to iron content and water quality in and around, Jagdalpur, Bastar district, Chattisgarh, India. J. Chem. Pharm. Res..

[41] Kumar V, Bharti PK, Talwar M, Tyagi AK, Kumar P (2017). Studies on high iron content in water resources of Moradabad district (UP), India. Water Sci..

[42] Asubiojo O (1997). Trace elements in drinking and groundwater samples in Southern Nigeria. Sci. Total Environ..

[43] Adebiyi F, Asubiojo O, Ajayi T, Obiajunwa E (2005). Trace element and physico-chemical characteristics of the sand and water fractions of Nigerian bituminous sands. Chem. Ecol..

[44] Jaishankar M, Tseten T, Anbalagan N, Mathew BB, Beeregowda KN (2014). Toxicity, mechanism and health effects of some heavy metals. Interdiscip. Toxicol..

[45] Pascual-Barrera A, Gold-Bouchot G, Ceja-Moreno V, del Río-García M (2004). Heavy metals and hydrocarbons in sediments from three lakes from San Miguel, Chiapas, México. Bull. Environ. Contam. Toxicol..

[46] Nkwunonwo UC, Odika PO, Onyia NI (2020). A review of the health implications of heavy metals in food chain in Nigeria. Sci. World J..

[47] Lateef T, Eluwole AB, Adewa DJ (2015). Geoelectrical assessment of the impact of the Ilokun dumpsite, Ado-Ekiti Southwestern Nigeria, on surrounding groundwater aquifers. Int. Lett. Nat. Sci..

[48] Ogundele LT, Adejoro IA, Ayeku PO (2019). Health risk assessment of heavy metals in soil samples from an abandoned industrial waste dumpsite in Ibadan, Nigeria. Environ. Monit. Assess..

[49] Ali IH (2021). Contamination and human health risk assessment of heavy metals in soil of a municipal solid waste dumpsite in Khamees-Mushait, Saudi Arabia. Toxin Rev..

[50] Wang Z-X (2014). Cadmium in agricultural soils, vegetables and rice and potential health risk in vicinity of Dabaoshan Mine in Shaoguan, China. J. Cent. South Univ..

[51] Li H, Qian X, Hu W, Wang Y, Gao H (2013). Chemical speciation and human health risk of trace metals in urban street dusts from a metropolitan city, Nanjing, SE China. Sci. Total Environ..

[52] Osipova NA, Filimonenko KA, Talovskaya AV, Yazikov EG (2015). Geochemical approach to human health risk assessment of inhaled trace elements in the vicinity of industrial enterprises in Tomsk, Russia. Hum. Ecol. Risk Assess. Int. J..

[53] Gu Y-G, Lin Q, Gao Y-P (2016). Metals in exposed-lawn soils from 18 urban parks and its human health implications in southern China's largest city, Guangzhou. J. Clean. Prod..

[54] EPAU (2001). Supplemental guidance for developing soil screening levels for superfund sites. Peer Rev. Draft OSWER.

[55] Adesida A, Anifowose AY, Ojo JS (2012). A study of basement fracture pattern around Akoko area of southwestern Nigeria for groundwater potential using high-resolution satellite imagery and electrical resistivity. Global J. Geol. Sci..

[56] Tosin AO, Ayokunle AA, Gbenga MO, Adebowale OA, Kola AA (2015). Geophysical and hydrochemical investigation of a municipal dumpsite in Ibadan, Southwest Nigeria. J. Environ. Earth Sci..

