# Supplementary material for: Geophysical and geochemical study of the contaminant impact of Oke-Tage solid waste dumpsite, Southwestern Nigeria
Source: Sci Rep. 2023 Mar 22;13:4704. doi: 10.1038/s41598-023-31948-3 (PMC10033965; doi:10.1038/s41598-023-31948-3)
Supplement: Supplementary file 1 — Supplementary Information. [file 41598_2023_31948_MOESM1_ESM.docx]

**Table S1. Calibration Parameters of AAS and % Recovery of Metals**

| Metal | Current (mA) | Wavelength (nm) | Calibration curve (R^2^) | Amount used for spiking (mg/kg) | Amount recovered (mg/kg) | Percentage recovery (% R) | LOD (mg/kg) | LOQ (mg/kg) |
| --- | --- | --- | --- | --- | --- | --- | --- | --- |
| Cu | 7 | 210.1 | 0.9965 | 20 | 19.62 | 98.10 | 0.01 | 0.03 |
| Cd | 8 | 228.6 | 0.9885 | 20 | 18.91 | 94.55 | 0.01 | 0.03 |
| Ni | 7 | 211.5 | 0.9979 | 20 | 17.63 | 88.15 | 0.05 | 0.05 |
| Fe | 6 | 325.1 | 0.9981 | 20 | 18.10 | 90.53 | 0.03 | 0.09 |
| Mn | 10 | 280.0 | 0.9930 | 20 | 16.93 | 84.65 | 0.005 | 0.015 |
| Pb | 10 | 282.9 | 0.9978 | 20 | 19.25 | 96.25 | 0.013 | 0.04 |
| Zn | 8 | 214.2 | 0.9976 | 20 | 19.87 | 99.35 | 0.005 | 0.015 |

**Table S2. One way analysis of variance showing metal variations among studied samples**

|  | | Sum of Squares | df | Mean Square | F | Sig. |
| --- | --- | --- | --- | --- | --- | --- |
| Cu | Between Groups | 0.065 | 1 | 0.065 | 0.382 | 0.564 |
|  | Within Groups | 0.844 | 5 | 0.169 |  |  |
|  | Total | 0.909 | 6 |  |  |  |
| Cd | Between Groups | 0.003 | 1 | 0.003 | 1.809 | 0.236 |
|  | Within Groups | 0.009 | 5 | 0.002 |  |  |
|  | Total | 0.012 | 6 |  |  |  |
| Pb | Between Groups | 0.002 | 1 | 0.002 | 1.638 | 0.257 |
|  | Within Groups | 0.006 | 5 | 0.001 |  |  |
|  | Total | 0.008 | 6 |  |  |  |
| Zn | Between Groups | 0.034 | 1 | 0.034 | 0.208 | 0.667 |
|  | Within Groups | 0.808 | 5 | 0.162 |  |  |
|  | Total | 0.842 | 6 |  |  |  |
| Fe | Between Groups | 0.004 | 1 | 0.004 | 2.920 | 0.148 |
|  | Within Groups | 0.008 | 5 | 0.002 |  |  |
|  | Total | 0.012 | 6 |  |  |  |
| Mn | Between Groups | 0.001 | 1 | 0.001 | 0.999 | 0.364 |
|  | Within Groups | 0.006 | 5 | 0.001 |  |  |
|  | Total | 0.007 | 6 |  |  |  |
| Mg | Between Groups | 0.002 | 1 | 0.002 | 1.142 | 0.334 |
|  | Within Groups | 0.011 | 5 | 0.002 |  |  |
|  | Total | 0.013 | 6 |  |  |  |
| K | Between Groups | 0.001 | 1 | 0.001 | 1.073 | 0.348 |
|  | Within Groups | 0.004 | 5 | 0.001 |  |  |
|  | Total | 0.005 | 6 |  |  |  |
| Na | Between Groups | 0.001 | 1 | 0.001 | 1.709 | 0.248 |
|  | Within Groups | 0.002 | 5 | 0.000 |  |  |
|  | Total | 0.003 | 6 |  |  |  |
| Ca | Between Groups | 0.003 | 1 | 0.003 | 1.547 | 0.269 |
|  | Within Groups | 0.010 | 5 | 0.002 |  |  |
|  | Total | 0.013 | 6 |  |  |  |
| Ni | Between Groups | 0.000 | 1 | 0.000 | 0.275 | 0.622 |
|  | Within Groups | 0.004 | 5 | 0.001 |  |  |
|  | Total | 0.004 | 6 |  |  |  |
